# Clinical Utility of Antithrombotic Prophylaxis in ART Procedures: An Italian Experience

**DOI:** 10.1371/journal.pone.0097604

**Published:** 2014-05-28

**Authors:** Elvira Grandone, Michela Villani, Giovanni L. Tiscia, Francesco Dentali, Donatella Colaizzo, Filomena Cappucci, Lucia Fischetti, Walter Ageno, Maurizio Margaglione

**Affiliations:** 1 Atherosclerosis and Thrombosis Unit, I.R.C.C.S. “Casa Sollievo della Sofferenza”, S. Giovanni Rotondo, Foggia, Italy; 2 Department of Clinical Medicine, Insubria University, Varese, Italy; 3 Medical Genetics, University of Foggia, Foggia, Italy; Medical Faculty, Otto-von-Guericke University Magdeburg, Medical Faculty, Germany

## Abstract

**Background:**

The usefulness of antithrombotic prophylaxis in management of Assisted Reproductive Technologies (ART) is questionable.

**Objectives:**

We prospectively examined the contribution of an antithrombotic prophylaxis in influencing clinical pregnancy and live-birth in an unselected cohort of women approaching ART.

**Patients/Methods:**

1107 women with fertility problems and a valid indication for ART were recruited. Baseline and follow-up information of obstetric outcomes and antithrombotic treatment were collected.

**Results and Conclusions:**

Median follow-up time was 34.5 months (range: 2–143). During the follow-up period, 595 (53.8%) women underwent ART (total 1234 cycles); 202 (33.9%) women achieved a pregnancy for a total of 255 clinical pregnancies. The concomitant use of LMWH and aspirin was significantly associated with a higher rate of clinical pregnancies (p: 0.003, OR: 4.9, 95% CI: 1.7–14.2). The pregnancy rate was also significantly increased by the use of LMWH alone (p: 0.005, OR: 2.6, 95% CI: 1.3–5.0). Carriership of inherited or acquired thrombophilia did not affect clinical outcomes of the ART. The efficacy of antithrombotic treatment was confirmed when the outcome “ live-birth” was considered. Present data suggest a potential benefit of antithrombotic prophylaxis during ART in improving the number of live-births.

## Introduction

Assisted reproductive technologies (ART) have been widely used in couples with fertility problems. However, the clinical pregnancy rate is low and results show a poorer perinatal outcome than non-assisted pregnancies [1].

Because of their antithrombotic and vasodilatory properties, many studies have investigated the effects of low-dose aspirin or low-molecular-weight heparin (LMWH) to improve ART outcomes. Recently, two systematic reviews and meta-analyses of the literature assessing the effect of low dose aspirin and LMWH in women undergoing ART showed that low dose aspirin was not associated with a higher rate of live birth, whereas LMWH could be effective in increasing the rate of live births [2], [3].

The biological plausibility of antithrombotic prophylaxis may be represented by a beneficial effect in counteracting existing or developing at risk pro-thrombotic conditions. However, data are controversial. A recently published systematic review and meta-analysis of the literature, including case-control studies, suggested that Factor V Leiden (FVL) is significantly associated with ART failure. Although the studies included were very heterogeneous, a positive association was found also for antiphospholipid antibodies. Conversely, neither prothrombin G20210A gene variant (PTm), nor protein S, protein C, or antithrombin deficiencies were associated with ART failure [4].

A recent Italian study found no statistical differences in the prevalence of thrombophilic mutations in women undergoing ART compared to women with spontaneous pregnancy [5]. Similarly, pregnancy outcomes and the risk of complications were not significantly different between carrier and non-mutation carrier women [5].

In a prospective cohort, we aimed at investigating the effect of antithrombotic prophylaxis on pregnancy outcomes (clinical pregnancies and delivery of a live newborn) after ART procedures. As secondary objective, the role of prothrombotic risk factors was assessed.

## Materials and Methods

### Participants

The entire cohort was previously described [6]. Briefly, between March 1998 and July 2011, a cohort of 1107 women from one Italian region (Apulia) approaching ART procedures were consecutively referred by local Fertility Clinics to our Thrombosis and Haemostasis Unit (I.R.C.C.S. “Casa Sollievo della Sofferenza”, S. Giovanni Rotondo, Italy). Baseline clinical information was collected. All women were investigated for the presence of inherited (FVL, PTm and deficiencies in protein S and C and antithrombin) and acquired thrombophilias (lupus anticoagulant, anticardiolipin antibodies).

One hundred and sixty-six (15%) women were lost to the follow-up; of the remaining, 595 women underwent at least 1 ART procedure. Pre-specified outcomes were the achievement of a “clinical pregnancy” (defined as the ultrasonographic visualization of one or more gestational sacs and foetal heart beat) [7], and the delivery of a live newborn (“live-birth”). In women who underwent ART procedures, clinical information on the use of antithrombotic prophylaxis, ART procedures and pregnancies were collected by a trained researcher. Follow-up information was obtained during the following check-up and/or by phone interviews. For each woman, the following information was collected for the analysis: number of cycles and type of treatment (embryo-transfer: in-vitro fertilization [IVF] and intra-cytoplasmatic sperm injection [ICSI], or intra-uterine injection [IUI]), prescription (yes/no), type (low-dose aspirin, LMWH or both), and duration of antithrombotic prophylaxis, and treatment outcomes. Only cycles starting with ovarian stimulation and ending with embryo-transfer/IUI were considered. The decision as to whether administer LMWH (enoxaparin or nadroparin at prophylactic doses: 4,000 IU and 3800 IU respectively, once per day by self-injection) and/or aspirin (100 mg, orally once daily) until the end of first trimester was entrusted to the attending physicians of the Fertility Clinics. Pregnancy loss was defined as a loss occurring before/at 20 weeks of gestation, whereas an Intra-Uterine Foetal death (IUFD) was a loss occurring after 20 weeks.

The study was approved by the IRB of “Casa Sollievo della Sofferenza”; participants gave their written informed consent for present and future use of the clinical data. The individual in this manuscript has given written informed consent (as outlined in PLOS consent form) to publish these case details.

### Laboratory tests

Blood samples were collected in 3.8% trisodium citrate and centrifuged at 2,000 *g* for 15 min to obtain platelet-poor plasma, that was frozen and stored in small aliquots at −70°C until tested. Antiphospholipid antibodies - lupus anticoagulant (LA) and IgG, IgM anticardiolipin antibodies (aCL) - antithrombin and protein C and total and free protein S were determined in all patients [8]. Cut-off for natural anticoagulants values were: 75–125% (antithrombin), 70–140% (Protein C) and 70–160% and 60–150% for total and free Protein S respectively. Severe thrombophilia was defined as the presence of natural anticoagulants deficiency or homozygosity for FVL or PTm, a confirmed presence of antiphospholipid antibodies, according to SSC Criteria [9], or the combination of more than one.

DNA was extracted from peripheral blood leukocytes according to standard protocols.

FVL and PTm genotyping was performed by a probe-based real time PCR technique [10].

### Statistical analysis

All the analyses were performed using SPSS version 11.0. The significance of any difference in proportions was tested using the Fisher exact test or by chi-square statistics as appropriate. Odds ratio (OR) and 95%-confidence intervals (CIs) were calculated.

The potential effect of the antithrombotic prophylaxis was assessed by multiple logistic regression analysis that controlled for potential confounding variables such as age, number of cycles, outcome of the first ART attempt, type of ART procedure (IUI or IVF/ICSI), the date (year) when each ART cycle was done, thrombophilias, and type of antithrombotic drug used. Conservative and optimistic live birth rates were calculated according to Malizia *et al*. [11]. Statistical significance was taken as p<0.05.

## Results

### Description of the cohort

**Figure 1:**
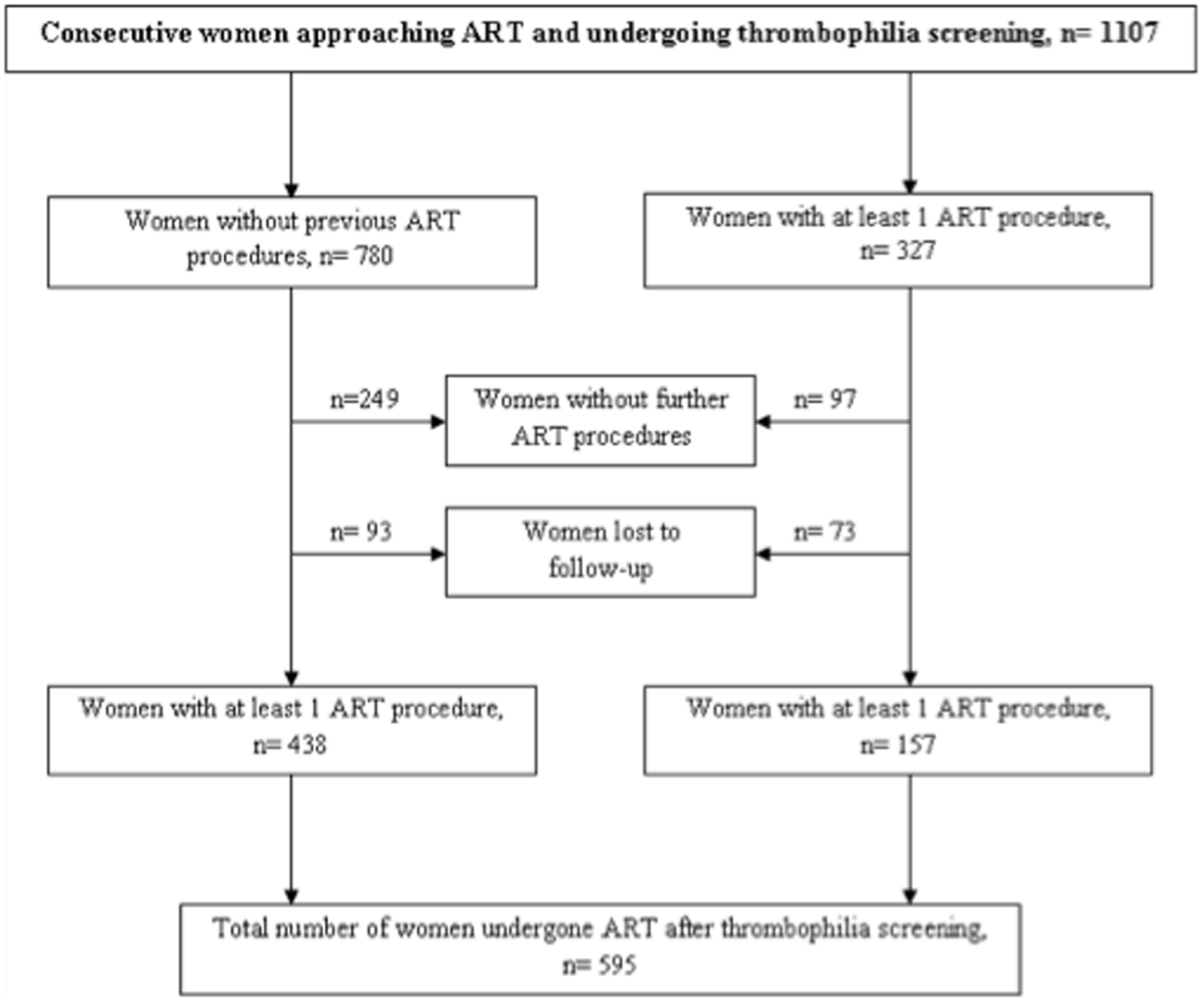
The figure depicts the entire cohort of women enrolled.

Flow diagram of enrolled women is reported in Figure 1. Overall, 1107 women were enrolled. Baseline characteristics are shown in Table 1. Median age was 35 yrs (range 18–49). Of the 337 previous natural conceptions, 208 (62%) resulted in spontaneous pregnancy loss. Prevalence of thrombophilic mutations was not significantly different from that reported in the general population [8]. In addition, inherited and acquired thrombophilias were not differently distributed according to causes of infertility (9.1% for the male factor, 8.2% for the pelvic/tubal factor, 11.6% in women with unexplained infertility and 12.3% in those observed for both male and female infertility, p: n.s.).

**Table 1:** Baseline characteristics and obstetric history of study participants (N = 1107).

Age [yrs], median (range)	35 (18–49)
**Smoking habits, n/N (%)**	**233/1107 (21)**
*1*–*10 cigarettes per day, n (%)*	*154 (66.1)*
*10*–*20 cigarettes per day, n (%)*	*61 (26.2)*
*> 20 cigarettes per day, n (%)*	*3 (1.3)*
* *Missing, n (%)*	*15 (6.4)*
**Infertility factors**	
*Male factor, n/N (%)*	*361/1107 (32.6)*
*Pelvic/Tubal factor, n/N (%)*	*243/1107 (21.9)*
*Unexplained, n/N (%)*	*335/1107 (30.3)*
*Mixed, n/N (%)*	*57/1107 (5.1)*
* *Unknown, n/N (%)*	*111/1107 (10.1)*
**Women with at least one natural conception, n/N (%)**	**173/1107 (15.6)**
*Natural conceptions, n*	*337* Δ
*Live births, n (%)*	*52 (15)*
Pregnancy losses, n (%)	*208 (62)*
**Women with at least 1 ART procedure, n/N (%)**	**327/1107 (29.5)**
*Type of ART procedure*	
*IUI, n (%)*	*64 (19.6)*
*IVF, n (%)*	*121 (37)*
*ICSI, n (%)*	*90 (27.5)*
*IUI+IVF+ICSI, n (%)*	*49 (15)*
* *Missing, n (%)*	*3(0.9)*
*Outcome of ART procedure, n*	*946*
*Clinical pregnancies, n (%)*	*131* □ *(13.8)*
*Live births, n (%)*	*21 (16)*
*Pregnancy losses, n (%)*	*103 (78.6)*
**FVL, n/N (%)**	**45/1107 (4)**
**PTm, n/N (%)**	**57/1107 (5)**
**Severe thrombophilias, n/N (%)**	**13/1107 (1)**

*Information not provided by couples

Δ 32 ectopic pregnancies, 1 ongoing pregnancy, 44 termination

□ 3 ectopic pregnancies, 3 ongoing pregnancy, 1 termination.

### Prospective analysis

Median follow-up time was 34.5 months (range: 2–143 months); 595 (53.8%) women underwent at least 1 ART procedure after the enrolment for a total of 1234 cycles.

Following an ART attempt, 202 (33.9%) women achieved at least a pregnancy for a total of 255 clinical pregnancies; of them 203 resulted in live-births.

One hundred eighty (30.3%) women were prescribed low-dose aspirin, for a total of 342 cycles (27.7%), whereas 46 women (7.7%) LMWH alone at prophylactic doses in 64 cycles (5.2%), and 12 women (2.0%) a combination of LMWH alone at prophylactic doses and low-dose aspirin in 16 cycles (1.3%) (Table 2). In order to investigate whether antithrombotic prophylaxis could influence the probability of achieving pre-specified outcomes, clinical pregnancies and live-births, reproductive outcomes following ART attempts were compared in treated and untreated cycles. Univariate analysis showed that antithrombotic prophylaxis with LMWH alone or combined with low-dose aspirin was significantly associated with both the pre-specified outcomes, “clinical pregnancy” (p: 0.001, OR 2.3, 95%CI 1.4–3.8) and “live-birth” (p: 0.001, OR 2.5, 95%CI 1.5–4.3). Antithrombotic prophylaxis with low-dose aspirin alone was not significantly associated with the “clinical pregnancy” (p: 0.8, Fisher exact test), or “live-birth” outcome (p: 0.4, Fisher exact test). Independently of the use of an antithrombotic prophylaxis, prevalence of total “clinical pregnancies” and “live-births” observed in thrombophilic and non thrombophilic women were not significant (data not shown). The logistic regression evaluated also a substantial difference in the prescription habits during all the years of observation showing no effect of the year in which ART cycles were carried out. In addition, the ORs for each type of treatment were not materially affected. The efficacy of LMWH on the number of live-births was confirmed when the analysis was restricted to women at first ART attempt (LMWH: p 0.02, OR: 4.2, 95%CI: 1.7–10.5).

**Table 2:** Main obstetric outcomes of cycles with and without medical treatment.

	Cycles (n)	Clinical pregnancies n (%)	Live- births n (%)
**ASA**	342	65 (19)	47 (13.7)
**LMWH**	64	20 (31.3)	19 (29.6)
**Combined treatment**	16	9 (56.3)	7 (43.8)
**No treatment**	812	161 (19.9)	130 (16.0)

Using a logistic regression analysis (Table 3), the concomitant use of LMWH and low-dose aspirin was significantly associated with a higher rate of “clinical pregnancies” (p: 0.001, OR: 5.3, 95% CI: 1.9–14.6). The pregnancy rate was also significantly increased by the use of LMWH alone (p: 0.03, OR: 1.9, 95% CI: 1.1–3.3). The efficacy of antithrombotic prophylaxis using LMWH, with or without low-dose aspirin, was confirmed when the pre-specified outcome “live-birth” was considered (Table 3).

**Table 3:** Clinical pregnancies and live births according to type of treatment in the prospective cohort. Logistic regression.

	Clinical pregnancies			Live- births		
	P-value	OR	95% CI	P-value	OR	95% CI
Age at screening	<0.001	0.9	0.9–0.96	<0.001	0.9	0.9–0.96
Treatment with ASA	0.12	1.4	0.9–2.0	0.32	1.2	0.8–1.9
Treatment with LMWH	0.005	2.6	1.3–5.0	0.002	2.9	1.5–5.7
Combined treatment	0.003	4.9	1.7–14.2	0.02	4.0	1.4–11.2

**Figure 2:**
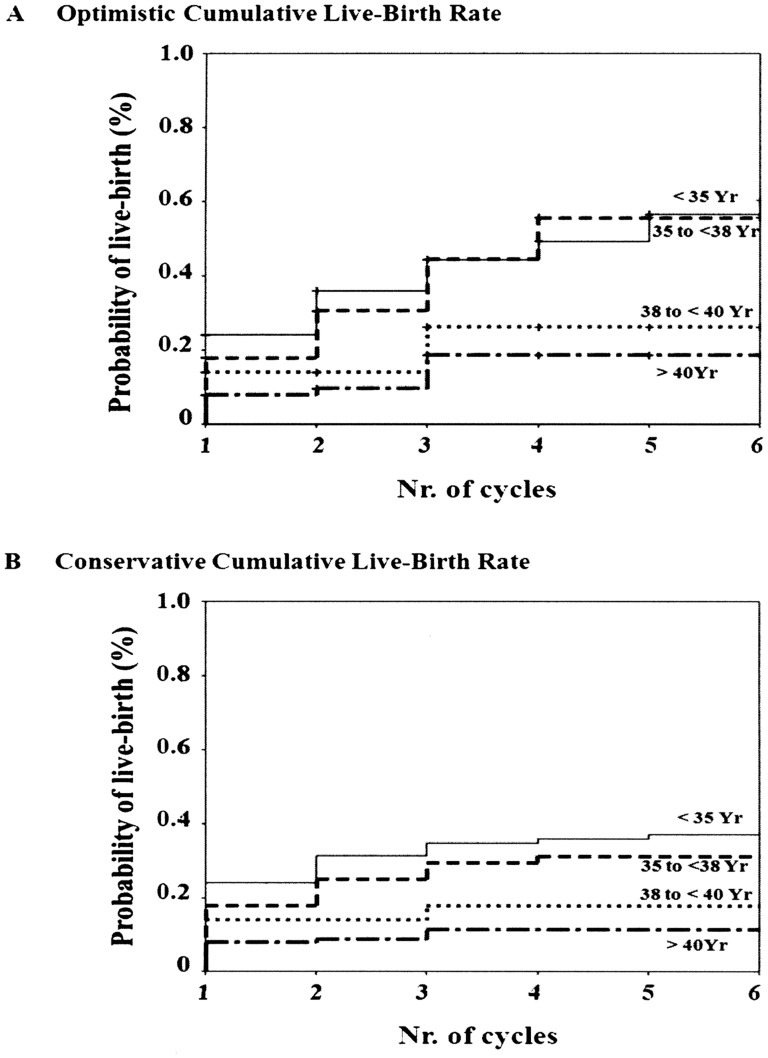
Cumulative live-birth rate during the follow-up and stratified according to the age, and calculated assuming that women who did not return for ART had the same chance as those who remained in treatment (Optimistic, Panel A) or no chance of a pregnancy resulting in a live-birth (Conservative, Panel B). In both models, the age-stratified curves in women from 38 to 40 yrs and >40 yrs were significantly different from those of women <35 yrs (p<0.001) and those in women from 35 to 38 yrs (p<0.01 and <0.05, respectively).

Younger age at enrolment was significantly and independently associated with both pre-specified outcomes, “clinical pregnancies” and “live-births” (Table 3). In keeping with this, both conservative and optimistic cumulative live-birth rate were significantly higher in younger women (Figure 2).

## Discussion

It was hypothesised that heparin can modulate many physiological processes required for blastocyst apposition, adherence and implantation with a potential role in improving pregnancy rates and outcomes [12]. However, available clinical data do not support the hypothesis that an antithrombotic prophylaxis could be useful in improving ART procedures [2], [3], [13]. A recent Cochrane review confirmed the lack of supportive data, identifying the need for adequately powered trials [13].

We followed-up a large cohort of infertile women approaching ART, who were prescribed antithrombotic therapy by Fertility Clinics according to local protocols. In this clinical setting investigated, an antithrombotic prophylaxis was prescribed in 422/1234 cycles (LMWH, low-dose of aspirin or both). Women who were prescribed prophylactic doses of LMWHs showed a significant improvement in terms of “clinical pregnancies” and “live-births”.

These data are in agreement with those from two recent systematic reviews and meta-analyses [2], [3], which demonstrated that LMWHs improve foetal outcomes in women undergoing ART, whereas the use of low-dose aspirin has no benefit.

Age was significantly and independently associated with both the pre-specified outcomes, “clinical pregnancy” and “live-birth”. We estimated cumulative live-birth rates according to Malizia and coll [11] who stratified data according to maternal age, then performed analyses using both optimistic and conservative methods. Optimistic methods assumed that patients who did not return for subsequent ART cycles would have the same chance of a pregnancy resulting in a live -birth as patients who continued treatment; conservative methods assumed no live- births among patients who did not return. In agreement with results from two large cohorts of women approaching ART procedures [11], [14], these data confirm that age is an important predictor of a successful outcome and strengthen the importance of conceiving at young age. Indeed, it is well known that complications of pregnancy increase for both the mother and the offspring with advanced maternal age [15]. Furthermore, age is a major concern in the available ART guidelines [16]–[18].

We observed a large cohort of infertile women (n = 1107) in a wide interval of time (more than 10 years), with a median of follow-up of 34 months. Because we enrolled all women observed in a wide range of time and in this large setting of women only a small group (n = 166, 15%) was lost to follow-up, a selection bias was unlikely.

We cannot exclude a “blurring” (contamination during the time due to some different attitudes in prescribing by physicians); however, we did not observe substantial differences during all the time of observation as far as the indication and the type of antithrombotic treatment.

Interviewer could have been influenced during the collection of data with special regard to treatment information. However, she did not know how the ART attempt ended and was unaware of the thrombophilia status.

In conclusion, present data suggest a potential benefit of antithrombotic prophylaxis and do not support the clinical utility of universal thrombophilia screening in improving the number of live-births.

Randomised controlled trials comparing different types of antithrombotic drugs during ART procedures are urgently needed.

## Supporting Information

Checklist S1 **STROBE Checklist.**
(DOC)
